# Supplementary material for: The interactome of intact mitochondria by cross-linking mass spectrometry provides evidence for coexisting respiratory supercomplexes
Source: Mol Cell Proteomics. 2017 Dec 8;17(2):216–32. doi: 10.1074/mcp.RA117.000470 (PMC5795388; doi:10.1074/mcp.RA117.000470)
Supplement: Supplemental Data [file supp_RA117.000470_133922_0_supp_23978_fzffwf.zip › spectra_annotation/mito_DR_spectra_annotation/232-2-5-1-1-1.pdf]

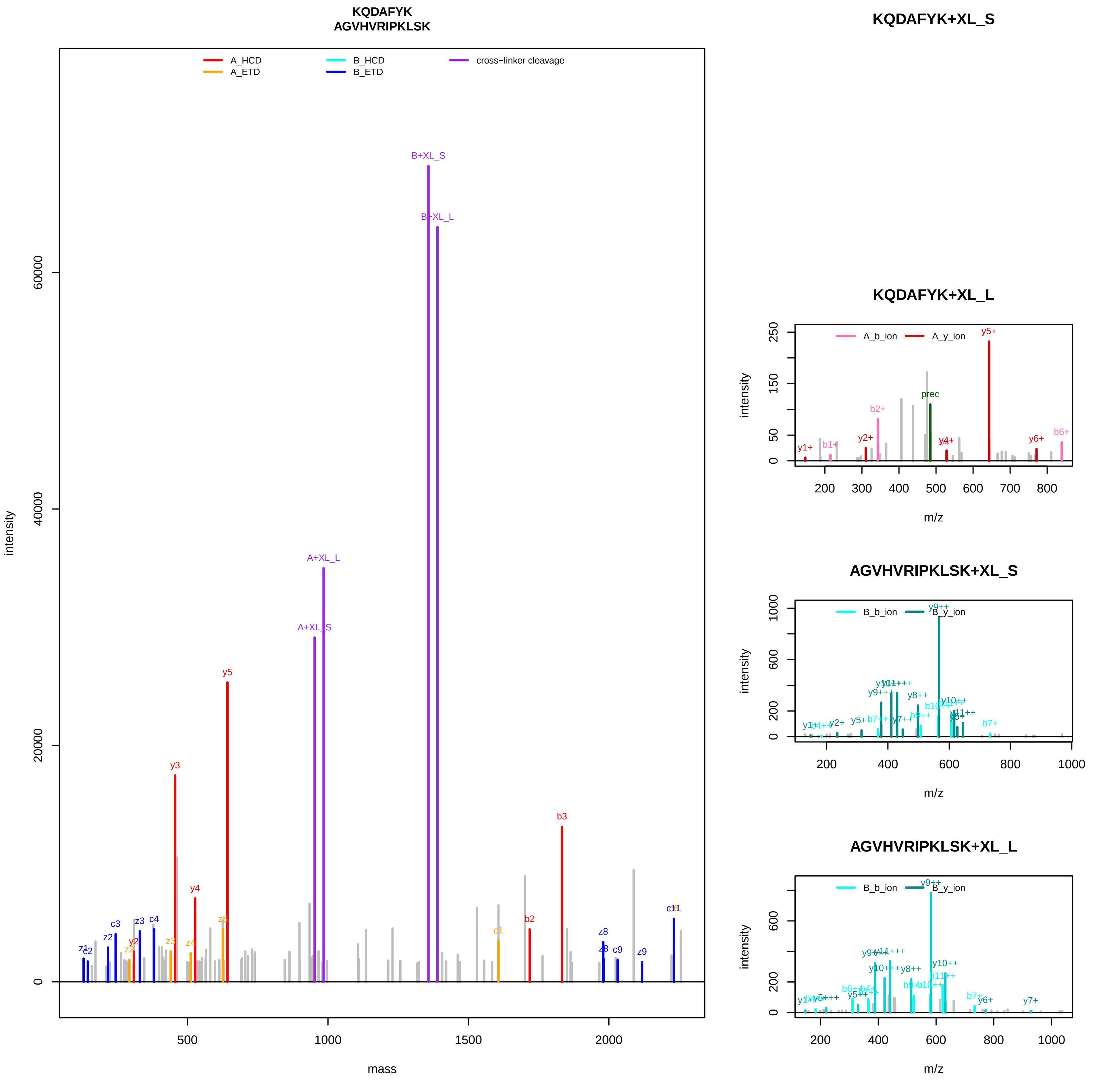
KQDAFYK
AGVHVRIPKLSK
A_HCD
A_ETD
B_HCD
B_ETD
cross−linker cleavage
B+XL_S
B+XL_L
A+XL_L
A+XL_S
y5
y3
y4
b3
b2
c1
z5
z4
z3
z2
y2
c3
z3
c4
z2
z1
c2
z8
z8
c9
z9
c11
intensity
mass
0
20000
40000
60000
500
1000
1500
2000
KQDAFYK+XL_S
KQDAFYK+XL_L
A_b_ion
A_y_ion
y5+
prec
b2+
y2+
y4+
y6+
b6+
y1+
b1+
m/z
AGVHVRIPKLSK+XL_S
B_b_ion
B_y_ion
y9++
y8++
y10++
y11++
b7+
y2+
y5+++
y1+
AGVHVRIPKLSK+XL_L
y10++
y8++
y6+
y7+
b7+
y1+
y5+++
